# NMR and MD Simulations of Non-Ionic Surfactants

**DOI:** 10.3390/molecules30020309

**Published:** 2025-01-14

**Authors:** Gerd Buntkowsky, Markus Hoffmann

**Affiliations:** 1 Department of Chemistry, Eduard-Zintl-Institute for Inorganic and Physical Chemistry, Technical University of Darmstadt, Peter-Grünberg-Straße 8, 64287 Darmstadt, Germany; 2 Department of Chemistry and Biochemistry, State University of New York Brockport, Brockport, NY 14420, USA

**Keywords:** surfactants, Nuclear Magnetic Resonance, molecular dynamics, confinement effects, host-guest interactions

## Abstract

Non-ionic surfactants are an important solvent in the field of green chemistry with tremendous application potential. Understanding their phase properties in bulk or in confined environments is of high commercial value. In recent years, the combination of molecular dynamics (MD) simulations with multinuclear solid-state NMR spectroscopy and calorimetric techniques has evolved into the most powerful tool for their investigation. Showing recent examples from our groups, the present review demonstrates the power and versatility of this approach, which can handle both small model-surfactants like octanol and large technical surfactants like technical polyethylene glycol (PEG) mixtures and reveals otherwise unobtainable knowledge about their phase behavior and the underlying molecular arrangements.

## 1. Introduction

Billions of tons of organic solvents are used every year in the fine chemical and pharmaceutical industries for reaction and product isolation processes. Because many organic solvents are problematic to human health and the environment due to their toxicity, volatility, flammability, and other hazardous characteristics, avoiding these or replacing them with innocuous ones is one of the 12 principles of Green Chemistry [1]. One possible solution to this problem is the use of surface activating agents, or surfactants for short.

Surfactants are molecules possessing an amphiphilic molecular structure. Besides their ability to lower the surface tension of water, hence their name, they easily adsorb to surfaces and aggregate themselves above a critical aggregate concentration. The literature related to the subject of surfactants is vast in both academic research as well as applied and industrial research. Besides their well-known use as detergents in soaps, personal care products [2,3,4], and cleaning products in general, [5] other applications include their use in the textile industry, [6,7] in lubricants [8] in paints, resins, and coatings, [7,9] pest control, [10] chromatography, [11,12] extraction, [13] and in pollution removal and prevention such as removal of metals, organic toxins, or oil spills [14]. Although they are essentially exclusively used as additives, their worldwide production as of 2024 exceeds 18 million tons [15]. There have been ongoing efforts to develop green surfactants as well as greener preparation methods for surfactants, as these developments are also driven by increasingly tighter environmental regulations [4,16]. Research efforts in this area can mainly be divided into deriving surfactants from natural sources [17,18,19,20,21,22,23,24,25,26,27], including their microbial production [28,29], and making them biodegradable [30,31].

**Figure 1:**
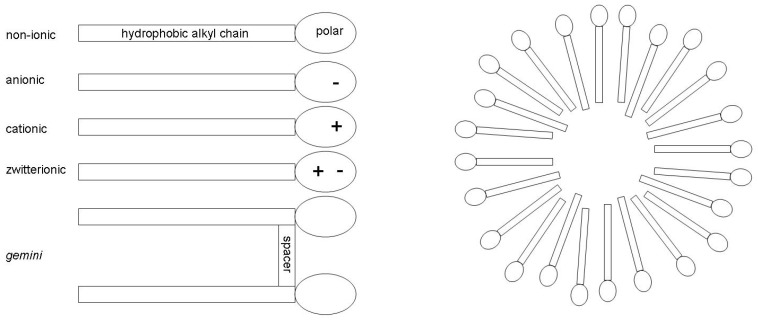
Schematic representation of the common types of surfactants (**left**) and scheme of micelle formation when the surfactant concentration, c, is greater than the critical micelle concentration, CMC.

As shown in the left panel of Figure 1, surfactants consist of a hydrophobic alkyl chain and a hydrophilic polar head group and can be separated into four different structural classes based on the type of the head group: non-ionic, anionic, cationic, or zwitterionic. The ionic surfactants require, of course, a counter-ion, usually inorganic in nature. In addition to these principal types of surfactants, there is also a more novel class of surfactants called *gemini* surfactants. Gemini surfactants may be viewed as dimeric surfactant molecules composed of two surfactant molecules that are linked together by some spacer chain, thus possessing two hydrophobic tails and two hydrophilic head groups. There are other substances, especially polymers, such as polyethylene glycol (PEG), which also display surface-activating properties. Although these substances lack a clear structural division of a hydrophobic moiety and a polar head group, they are often also referred to as surfactants.

Because of its hydrophobic structural moiety, when a surfactant molecule is added to water, it preferentially resides at the air–water interface, which substantially reduces its surface tension. For biphasic oil–water systems, surfactants tend to reside at the oil–water interface and greatly aid in stabilizing oil–water emulsions. When a surfactant is added to water in sufficient amounts, exceeding the so-called critical micelle concentration (CMC), the air–water interface is saturated with surfactant molecules, and the surfactants self-assemble into aggregates known as micelles, which are dissolved into the bulk water (see right panel of Figure 1). The presence of micelles greatly increases the solubility of non-polar organic substances compared to pure water. These principle self-aggregation processes of surfactants lay the foundation for their use as detergents, emulsifiers, foam stabilizers, and wetting agents and have thus been a continued subject of intense research in both academia and industry.

A typical aggregation number of surfactant molecules in a micelle is about 50 [32]. The cohesion of the surfactant molecules in the micelle is through non-covalent forces. While the non-polar alkyl chains attract one another through hydrophobic interactions, the polar head groups repel each other, especially in the case of ionic surfactants. One uses the hydrophilic–lipophilic balance (HLB), which can be calculated in increments, to characterize surfactants. Another parameter that characterizes the surfactant performance is the CMC, where a lower CMC indicates that less surfactant is required for a particular application. The CMC usually decreases with increasing the alkyl chain length of the hydrophobic moiety of the surfactant molecule, and a minimum alkyl chain length of at least about 10 carbon atoms is usually needed for micelle formation to occur. For perfluorinated alkyl chains, which are more hydrophobic, the minimum chain length for micelle formation can even be shorter.

In the case of ionic surfactants, the role of the counter-ions is also very important for the structural balance of the micellar aggregates. Seventy to ninety percent of the counter-ions typically reside in the Stern-Layer, i.e., in the immediate vicinity of the micelles. Thus, the degree of association is typically rather high, and only a minor fraction of dissociated counter-ions resides in the next nearest layer, known as the Guy–Chapman layer. It is also important to keep in mind that micelle formation is a highly dynamic process. Rapid exchange of surfactant molecules with surfactant molecules freely dissolved in the bulk water can occur. In fact, the entire micelles may form and decay within milliseconds [33].

Micelle formation can also occur in non-polar media. Here, the surfactants form a reverse micelle where the polar head groups are pointed inward, and the hydrophobic tails point outward to interact favorably with the non-polar solvent. Simply put, just like oil can be emulsified in water via micelle formation, water can be emulsified in oil via reverse micelle formation. An often-used shorthand notation to distinguish between these two types of emulsions is *o*/*w* for oil-in-water emulsion and *w*/*o* for water-in-oil emulsion.

The thermodynamics and phase behavior of binary surfactants and water systems are quite intriguing and complex. The micelle formation phenomenon occurs only if the temperature is above a system-dependent minimum temperature, referred to as the Krafft temperature. In turn, for non-ionic surfactants, a mono-phasic micellar solution will reach a cloud point upon heating, separating into a water-rich and a surfactant-rich phase. Furthermore, which is often overlooked, there is a fascinating extraordinary variety of phase behavior at surfactant-rich concentrations, which are characterized by different aggregate structures such as rod-like, hexagonal, or lamellar. The interested reader is referred to several excellent reviews on this subject [34,35,36].

The presence of micelles usually dramatically increases the solubility up to a “maximum additive concentration” (MAC) of hydrophobic molecules in water. This effect is commonly referred to as solubilization. Thermodynamically stable microemulsions may be formed when more compound than the MAC is added and a new phase is formed. Since microemulsions are usually optically transparent because of the small size of the emulsion droplets, the formally biphasic system is often regarded as being in a single phase. In any event, even when droplets are much larger, and the system is visibly a biphasic emulsion, i.e., a colloidal system, the surfactant stabilizes the emulsion to remain suspended for very long times, which is important for applications such as emulsion polymerization or the formulations of paints, cements, or cosmetics.

For synthetic chemists, surfactants play an important role in opening up possibilities for chemical synthesis in water as an environmentally friendly solvent. After initial works by the Breslow group in the early 1980s demonstrated that water can, in contrast to prior wisdom, be an effective solvent in organic synthesis, [37] surfactants were used to increase solubility and provide dispersed interface-rich systems. Moreover, the core of the oil-in-water microemulsion formed by surfactants constitutes an interesting reaction medium that may, in some cases, lead to accelerated reactions or even altered reaction path ways [38]. In part, the reasons for these synthesis advantages are the reactants being forced into close proximity within the confinement of such microenvironments [39]. Such soft confinement environments have been the subject of significant research, and reviews on various aspects of this topic have appeared [40,41,42,43]. This review does cover some examples of soft confinement environments but mainly focuses on systems where the surfactants themselves are confined within a hard, i.e., solid confinement environment such as mesoporous high surface materials. In this respect, surfactants also play a crucial role as templating agents in many synthesis procedures of high-surface materials. This aspect is outside the scope of this review, and the interested reader is referred to several available reviews [44,45,46,47].

**Figure 2:**
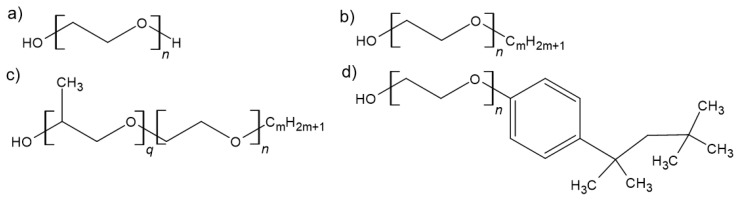
Chemical structures of Surfactants: (**a**) PEG, (**b**) C_m_E_n_, (**c**) C_m_E_n_P_q_, and (**d**) Triton X-100. For the surfactants, m indicates the number of carbon atoms in the alkyl chain (C), while n and *q* are the repetition units of ethylene oxide (E) and propylene glycol (P), respectively.

Polyethylene glycol (PEG, Figure 2a) has been increasingly used as a very attractive alternative solvent for Green Chemistry because it possesses a negligible vapor pressure, is nontoxic, biodegradable, and inexpensively available since it is annually produced at about 600,000 tons, primarily for the medical and personal care industries [48]. Advances in using PEG as a solvent have been reviewed with increasing frequency [49,50,51,52], and the need for physicochemical studies to elucidate the role of PEG as a chemical solvent has been noted. Briefly, the main reasons for the successful use of PEG as a reaction medium include its ability to dissolve a wide range of substances, including some mineral salts, that it is a suitable solvent for microwave-assisted synthesis, and that, in some cases, may act as acid-base or redox catalyst. The latter is important for transition metal-catalyzed reactions, including heterogeneously catalyzed reactions where the transition metal catalyst is immobilized on a solid material of a large surface area [53]. In addition to PEG, non-ionic surfactants such as C_m_E_n_, as defined in Figure 1, are related to PEG and possess similar physical and environmentally benign characteristics [54]. Their amphiphilic nature increases the range of chemicals they can dissolve, as illustrated by the successfully carried out Diels–Alder reaction in C_6_E_10_ as solvent [48]. Therefore, liquid non-ionic surfactants present another group that has not yet been recognized as a neoteric solvent. A number of experimental studies have shown that the absorption of C_m_E_n_ non-ionic surfactants from aqueous solutions in hydrophilic mesoporous silica strongly depends on the surface concentration, changing adsorption from small patches to micelles to worm-like aggregates and eventually to bilayer formation [55,56,57,58,59].

The interest in advancing knowledge about PEG and C_m_E_n_ is largely driven by the fact that they are very attractive solvent media for catalyzed reactions. For example, palladium-catalyzed reactions are of particularly wide interest in the fine chemical and pharmaceutical industry because they allow for the direct formation of carbon-carbon single bonds, which generally is very difficult to achieve. Heck, Negishi, and Suzuki were awarded a Nobel Prize in Chemistry in 2010 for their pioneering work on Palladium catalysis [60]. Initially, only homogeneous Pd catalysts were used until 1999, when Wall and coworkers reported for the first time heterogeneous Pd catalysis [61]. Heterogeneous catalysts are advantageous because of their facilitated recovery and avoidance of transition metal catalyst impurities in the synthesis product. As recently reviewed, [62,63] to date, there is a large variety of heterogeneous catalysts available. These vary in their type of support materials, which also include mesoporous silica materials, as first reported by Jana et al. [64]. Recent developments in Pd-catalyzed reactions include efforts to avoid toxic solvents either by solventless approaches [65] or by the use of green solvents such as water [62] and PEG [66], where PEG was also successfully used as a reaction medium in heterogeneously Pd-catalysis with Pd immobilized on mesoporous solid support [67]. There is also considerable research on determining mechanistic details of heterogeneously Pd-catalyzed reactions, as has been carefully summarized [60]. Many factors, including the solvent medium and the chemistry of the support surface and of the reactants, have an influence on the reaction mechanism. Interestingly, to the best of our knowledge, possible confinement effects due to small pore sizes of the mesoporous support material have not been considered yet. These could be significant because it is known that many small molecules exhibit very different physicochemical properties in confinement compared to their bulk phase, as has been reviewed [68]. For example, it has been demonstrated that the dielectric constant of water drastically reduces under confinement [69].

Commercial PEGs are mixtures of ethylene glycol oligomers/polymers where the average molar weight is described by the product name. For example, PEG1000 has an average molar weight of about 1000 g·mol^−1^. PEGs with an average molar weight of 800 g·mol^−1^ and lower are liquid at room temperature and have been successfully used in chemical synthesis [49,50,51].

Owing to the potential of liquid non-ionic C_m_E_n_ PEG surfactants as environmentally benign solvents in heterogeneous catalysis, where the solvent experiences confinement effects, there was a pressing need to understand confinement effects for PEG and C_n_E_m_ residing in pore structures relevant for immobilized transition metal catalysts to advance their effective use as an environmentally benign solvent medium in heterogeneous Pd-catalysis. (as details on catalysis are beyond the scope of this review, we point the reader to a recent overview article [63] and references therein).

To achieve a better understanding of PEG and related non-ionic surfactants under confinement, it is necessary to employ analytical techniques that can reveal structural features on the molecular length scales while not depending on the local or full crystallinity of the material. Solid state NMR spectroscopy (SSNMR) is an analytical technique for the characterization of the structure and dynamics of solid materials. It analyzes local interactions between the NMR active nucleus and its direct surroundings at the Angstrom to Nanometer scale. In contrast to diffraction techniques, its applicability is independent of the presence of long-range ordering or crystallinity. Thus, it is particularly well suited for the investigation of structurally disordered systems, such as molecules in confinement [70]. SSNMR can analyze the structure and dynamics of molecules in confinement and is capable of directly probing the properties of surface-adsorbed molecules and their interactions with the confining surfaces. As SSNMR investigates short-range interactions, it is essential to combine it with a computational modeling technique, which can convert the local structural information into structures of larger aggregates with respect to local structural ordering. As the structures of interest are too big to employ quantum chemical techniques such as density functional theory (DFT), Molecular Dynamics (MD) simulations are currently the most powerful simulation techniques. Accordingly, in the last decade, the combination of MD simulations and variable temperature ssNMR has evolved into a powerful tool to characterize the phase behavior of small solvent or probe molecules in confined phases, such as, e.g., porous silica or carbon or materials in general and specifically of non-ionic surfactants in confined systems (see, e.g., recent reviews, [71,72,73,74] and references therein).

A detailed introduction to the basics and techniques of SSNMR, time-resolved NMR, and MD techniques is beyond the scope of the present review. Here, the reader is referred to recent review articles (see, e.g., [75,76,77] or [78] or [79,80] and references therein) or the encyclopedia of magnetic resonance. Here, only some salient features are briefly summarized.

SSNMR probes local interactions, such as the chemical shift, which is a measure of the influence of the local chemical environment on the NMR nucleus, the magnetic dipole-dipole interaction, which measures the distance between two NMR active nuclei, and, in the case of quadrupolar nuclei with spin >1/2, the changes of the electric field (electric field gradient, EFG) at the position of the nucleus (nuclear quadrupole interaction). In particular, for covalently bonded deuterons in organic molecules, where the electric field is very strongly connected to the bond directions, the quadrupolar interaction is an extremely sensitive reporter of rotational or vibrational molecular dynamics.

For analytical studies, ssNMR experiments are generally performed under magic angle sample spinning (MAS [81,82]) conditions, where the sample is fast spun around an axis of 54.7° to achieve chemical shift resolved spectra. For spin-1/2 X-nuclei such as ^13^C or ^15^N, the sensitivity of MAS NMR is often increased by cross-polarization from protons (CPMAS) [83,84]. For structural studies, MAS experiments are often combined with dipolar recoupling techniques, which selectively reintroduce the magnetic dipolar interaction between spins in MAS experiments [85,86,87,88]. By suitable choice of variable temperature SSNMR techniques, it is possible to cover a wide dynamic range of physical and chemical processes inside materials, spanning timescales from 10^+7^ sec down to 10^−12^ sec. Further sensitivity enhancement by one to two orders of magnitude of NMR is achievable by high-field Dynamic Nuclear Polarization (solid-state DNP) [89,90,91]. However, as spectrometers are very costly, they are available only in a few laboratories worldwide.

MD simulations, which are based on molecular force-fields and classical dynamics, can give access to information such as density profiles across pore diameters that are experimentally very difficult, if not impossible, to determine [73] and, in this way, can assist in the molecular-level interpretation of ssNMR data [92]. However, combined ssNMR and MD simulation studies are exceedingly rare [93]. In general, available studies on the confinement of PEG and C_m_E_n_ surfactants are rather limited and are briefly summarized here. Sun et al. [94] studied the intrusion and extrusion processes of PEG, water, and their mixtures into silica pores as a function of average pore size. While neat water requires the application of external pressure, PEG intruded spontaneously into the silica pores. The authors could infer from their data that the structural configuration of the confined PEG differs from that of bulk PEG. In another study, PEG1500 was observed to crystallize in a concentrical lamellar form in bulk and under confinement in large pores, but the crystals changed to an unbent lamellar form when the pore size was smaller than 71.1 nm [95].

In the following, an overview of recent studies of our groups, where conventional SSNMR, DNP-enhanced DNP-SSNMR, and MD simulations are used to shed light on the properties of confined surfactants, is given. The examples range from small model surfactants to technical PEG mixtures

## 2. Typical Results

In the following sections, we first present recent examples of small model surfactants in confinement and then examples of real PEGs and other complex surfactants in confinement are reviewed.

## 3. Small Molecules as Models for Confined Surfactants

1-Octanol and isobutyric acid (iBA, 2-methylpropanoic acid) and their aqueous solutions were employed as small molecular models of larger surfactants in a number of NMR and molecular dynamics investigations. Similar to larger surfactants, they are of an amphiphilic nature with a hydrophilic head and a hydrophobic tail group. Combined as binary mixtures with water, they are models for many natural or technical systems, such as micelles, vesicles, cell walls, or natural membranes [96]. The water octanol partition coefficient or *p*-value K_ow_ is the industry reference standard for the quantitative description of the phase properties of immiscible water/oil mixtures, owing to the large miscibility gap of water octanol mixtures [97]. Liquids with low K_ow_ are hydrophilic, and liquids with high K_ow_ are hydrophobic. The K_ow_-values are important numbers, e.g., in pharmacology, to predict the distribution of drugs within a body [98]. In contrast to octanol, above a concentration-dependent critical temperature, water and iBA are completely miscible. Below this temperature, water and an iBA-rich fraction coexist.

### 3.1. Water–Octanol Mixtures

Multinuclear NMR under static and MAS conditions is an ideal tool to investigate the structure and dynamics of confined guest molecules. While interactions of spin-1/2 nuclei such as ^1^H, ^13^C, or ^29^Si chemical shift and dipolar interactions are sensitive to local structures and molecular ordering and arrangement, quadrupolar interactions of ^2^H-nuclei respond strongly to local mobility and dynamics. In order to demonstrate this, Figure 2 displays the results of an experimental SSNMR study of water/octanol mixtures confined in mesoporous SBA-15 in order to study their local structure. Employing a combination of 1D ^1^H-MAS NMR, ^29^Si{^1^H}-CPMAS (Cross-Polarization Magic Angle Spinning) NMR, and ^1^H/^29^Si-HETCOR (Heteronuclear Correlation) experiments, obtained by Frequency Switched Lee-Goldburg (FSLG)-NMR, [99]. Variation of the contact time in these experiments allowed us to gain a sense of how close various moieties of the confined solvent molecules are to the pore surface and thus about the local structures and thus determine the distributions of the two liquids inside the confinement [52]. Effects of the sample spinning on the confined liquids can be neglected at the employed spinning frequencies.

**Figure 3:**
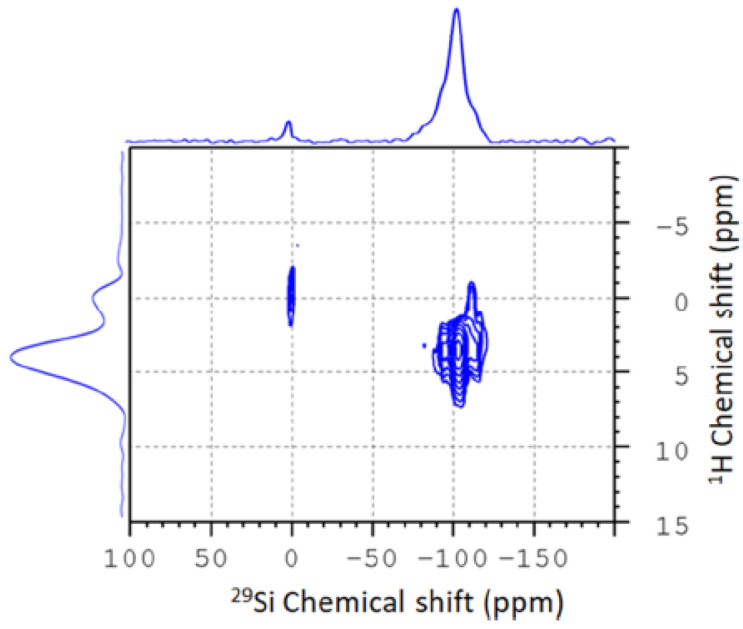
Low-temperature ^1^H/^29^Si CP-MAS FSLG HETCOR obtained with 9 ms contact time of an 80:20 mol% mixture 1-octanol:water mixture, depicted by Kumari et al. [52]. Reprinted with permission from Kumari et al. [52]. Copyright 2018 American Chemical Society.

These studies illustrate the effective use of ^1^H/^13^C CP-MAS FSLG HETCOR experiments to elucidate the orientation of confined molecules relative to the surface of porous silica, as included in Figure 3 (left, middle). In this context, we have recently developed a convenient approach for chemical shift referencing the ^1^H dimension in FSLG HETCOR experiments (from Kumari et al. [52]).

**Figure 4:**
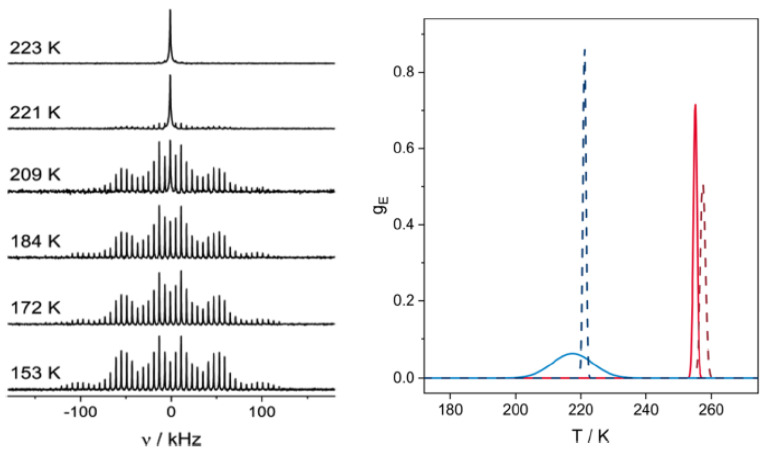
Selected temperature-dependent ^2^H MAS NMR spectra (left) of 1-octanol-d17 confined in mesoporous SBA-15 (illustrated, middle). All spectra are normalized to an equal height. Changes in the line shapes reflect the motional dynamics affected by the liquid-to-solid phase transitions whose distribution of activation energies, g_E_ (right panel), become visibly broader under confinement (blue) compared to bulk (red) for the data obtained from MAS conditions (solid line) compared to static conditions (dashed line) (see ref. [100] for details). Reprinted with permission from Döller et al. [100] Copyright 2021 American Chemical Society.

The interaction of the quadrupole moment of the ^2^H nucleus with the electric field gradient produced from any uneven charge distribution in its surroundings is highly sensitive to dynamic processes, which is directly observable by the resulting line shape of the ^2^H ssNMR signal. Consequently, ^2^H ssNMR spectra provide information about chemical binding details, rotational and translational movements, or sorption kinetics specific to the probed compound [68]. Building on prior work on 1-octanol, [52] we first developed a convenient method for chemical shift referencing in two-dimensional (2D) spectra [99] and then moved on to the study of deuterated 1-octanol-d17 [100]. The ^2^H MAS NMR and static ^2^H NMR spectra of 1-octanol-d17 in SBA-15 confinement were recorded as a function of temperature (see Figure 4) and compared with analogous spectra obtained from bulk, unconfined 1-octanol-d17. Careful line shape analysis of the spectral data revealed that the melting processes of confined 1-octanol-d17 are slowed, exhibiting a broad non-Gaussian distribution of activation energies while forming a pore solid with smaller crystallite sizes as in unconfined 1-octanol-d17.

**Figure 5:**
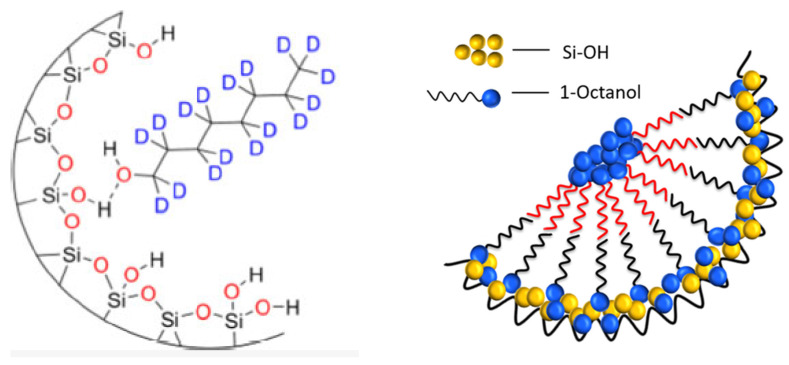
Hydrogen bonding of octanol and the silica surface orients the octanol molecules (**left**). By a combination of hydrophilic and hydrophobic interactions, this ordering is transmitted inside the pores (**right**), creating a cylindrical double-layer structure (for details, see Kumari et al. [52]). Reprinted with permission from Kumari et al. [52] and Döller et al. [100]. Copyright 2018 and 2021 American Chemical Society.

From the combination of solid-state NMR and MD simulations, a model of the packing of the octanol molecules inside the pores is deduced, as sketched in Figure 5. The hydrogen bonding between the hydroxy groups of octanol and surface silanol-groups causes an ordering of the octanol molecules on the surface, which is transmitted via a combination of hydrophilic and hydrophobic interactions inside the pores, resulting in a cylindrical double-layer structure. This is in contrast to smaller molecules like water, [74,101,102,103,104,105] benzene [106,107] or ethylene-glycol [108,109], which exhibit coexistence between disordered surface phases on the pore walls and crystalline inner phases in the center of the pores.

### 3.2. Water–Isobutyric Acid Mixtures

**Figure 6:**
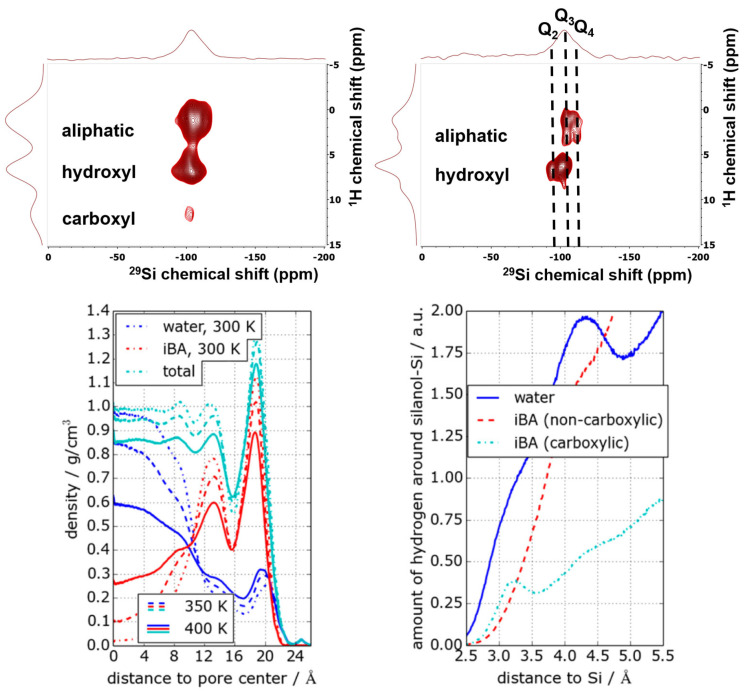
Solid-state NMR and MD simulations of water -isobutyric acid mixtures (56 wt% iBA) confined in SBA-15 Upper row: 2D ^1^H-^29^Si FSLG HETCOR at 9.4Tesla, 110 K and 8 kHz MAS. The spectrum measured with a long contact time of 3 ms (**left**) reveals cross-peaks from silica to carboxyl, hydroxyl, and aliphatic protons. At short contact times (**right**) of 0.5 ms, only cross peaks to aliphatic and hydroxyl-protons are visible. Lower row: density profiles for iBA and water (**left**) and hydrogen atom density (**right**). The pore center is at 0 Å, and the pore wall is at 25 Å. Reprinted with permission from Harrach et al. [92]. Copyright 2015 American Chemical Society.

As already mentioned in the introduction of this section, bulk mixtures of iBA and water exhibit a richer phase behavior than water/octanol mixtures. Owing to this richness, they were employed as model systems for the investigation of the basic processes of microphase separations in small confinement. Employing a combination of ^1^H-diffusometry and ^1^H-relaxometry, an anomalous temperature dependence of the self-diffusion coefficient and a bifurcation of the T_2_-relaxation upon a critical temperature of 42 °C was observed in confinement [110,111,112]. The detailed analysis of these data revealed the presence of cylindrical liquid layers. The assignment of these layers to water and an iBA reach phase was achieved by a combination of ^1^H-^29^Si FSLG HETCOR and MD simulations on frozen solutions (110 K) [92]. The slowing of the molecular dynamics at those low temperatures causes an incomplete averaging of the magnetic dipolar interactions of the confined molecules, resulting in cross-peaks between different proton species and the surface silica groups in the HETCOR spectra. Different distance ranges can be mapped out by a variation of the contact time in the HETCOR experiments (Figure 6, upper panel). The MD simulations reveal the distribution of the phases inside the pores as density profiles (Figure 6, lower panel) of water and iBA as a function of the distance from the pore center. They corroborate the cylindrical model and assign the water-rich phase to the inner cylinder and the iBA-rich phase to the outer cylinder, where the iBA molecules orient preferentially like an inverted brush-like structure. This orientation is caused by hydrogen bonding interactions between surface silanol groups and the carboxylic acid groups of the iBA. As a result of this, the aliphatic chains are oriented toward the pore center [92]. Thus, despite the chemical differences between octanol and iBAs, they exhibit similar structural behavior in the confinement. An interesting result from the detailed analysis of the MD simulations was that the phase-behavior at low temperatures is mainly caused by the hydrogen-bonding enthalpy. Increasing the temperature causes a strengthening of entropic terms, which creates stronger disorder and results in higher miscibility (see [92] for details).

### 3.3. Confined Surfactants

This section reviews studies of non-ionic surfactants confined in aminopropyltriethoxysilane (APTES) modified SBA-15 [113] studied by a combination of DSC, SSNMR, and DNP [114,115,116,117,118].

**Figure 7:**
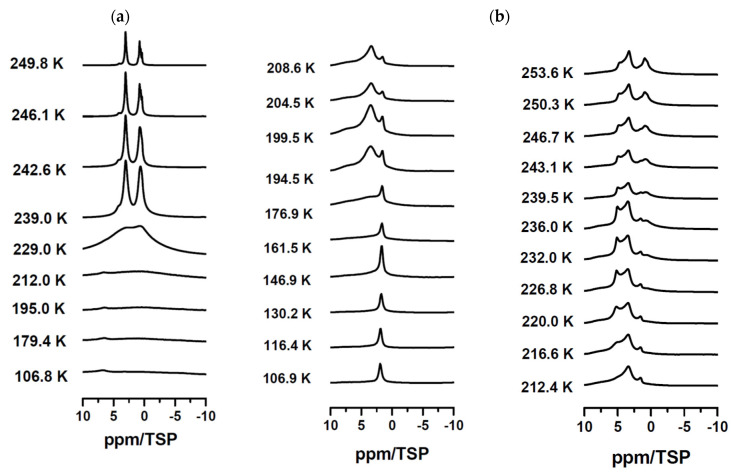
Temperature-dependent ^1^H MAS spectra [93] of (**a**) neat C_10_E_6_P_2_ and (**b**) C_10_E_6_P_2_ confined in mesoporous silica material measured at 5 kHz spinning in the range between 106.9 K and 253.6 K.

The temperature-dependent ^1^H-MAS-NMR (Figure 7a) of bulk C_10_E_6_P_2_ at low temperatures (106–212 K) are extremely broadened by homonuclear proton dipolar interactions, showing their very slow molecular dynamics, which is in the rigid limit for NMR. Above 220 K, a narrowing of the lines is observed, showing the onset of fast molecular motions, which continues upon further increase of the temperature, resulting in a chemical-shift resolved spectra of the molten surfactant with relatively narrow lines above 239 K. In contrast, the spectra of confined C_10_E_6_P_2_ present a richer behavior (Figure 7b). Already at 106 K, the spectrum consists of a narrow signal and a very strongly dipolar broadened background signal. The narrow signal was attributed to surface Si-OH groups, and the broad signals to the confined surfactant [93]. Already at temperatures above 176.9 K, the broadened background signal starts to narrow, showing that due to the confinement, the melting process inside the pores starts approximately 40 K below the melting temperature of the bulk surfactant.

Further insight into the structures of the confined surfactants is gained by DNP-enhanced solid-state NMR spectroscopy. Owing to the large signal enhancement by DNP, it is possible to measure, e.g., the natural abundance of 13C-SSNMR spectra of the confined surfactants in a very short time, compared to regular SSNMR. Moreover, as the radical influence the NMR properties of its environment (see, e.g., review [91] and references therein), when we started to employ DNP on PEG, we had the intention to investigate intermolecular interactions between confined surfactant molecules and the DNP radical, which is used as the polarization source in DNP experiments. In these experiments, we found the puzzling result that DNP enhanced ^13^C Magic Angle Spinning (MAS) spectra of the surfactant consist of a superposition of two different sub-spectra that is 180° opposite in phase. One of those had relatively broad and the other fairly sharp spectral features [119]. A detailed analysis of the effect showed that it is caused by the simultaneous presence of two independent polarization channels. By Hahn-echo experiments, we could show that the broad sub-spectrum stem from surfactant molecules in the coordinate spheres of the DNP radical and the sharp features from distant surfactant molecules, which are polarized by a combination of proton spin diffusion and nuclear Overhauser effect (NOE) [119,120]. Moreover, we could show that it is possible to select the effective polarization channel by manipulating the proton spin reservoir via simple trains of 180°-pulses on the proton channel. With these results, we developed a DNP strategy to selectively detect molecules in the coordination sphere of the radical, polarized via the direct channel, or alternatively far away from the radical, polarized via the indirect channel. In order to extend the application potential of this new technique, we did a comparative study of the magnetic field dependence employed in the non-ionic surfactant C_10_E_6_, employing AMUPol as a DNP radical [121]. This study revealed the nature of the dominant polarization transfer mechanism of the direct channel as a cross-effect and showed that the relaxation effects of methyl groups are important for the efficacy of the indirect channel. Finally, the analysis of the direct channel resonances indicated the preferential structural orientation of the polar ethylene oxide head group of C_10_E_6_ towards the AMUPol radical.

A direct application of this new technique was the detection of unusual local molecular motions of the surfactants in the solid state [122]. This detection was based on the fact that the presence of NOEs is an indicator of modulations of local fields caused by motional processes. This revealed the interesting results that in all studied surfactants (PEG 200, C11E6P1, C10E6, Triton X-100, and solutions of organic solutes in PEG 200), local molecular motions beyond the generally known rapid rotation of methyl groups must be active even at low temperatures of 117 K. DSC investigations of the samples showed that these local molecular motions are present not in glass-forming systems, but also in systems that ordinarily tend to crystallize. This indicated that the rapid cooling that the sample experiences upon introduction into the DNP NMR probe may at least partially preserve local mobility.

**Figure 8:**
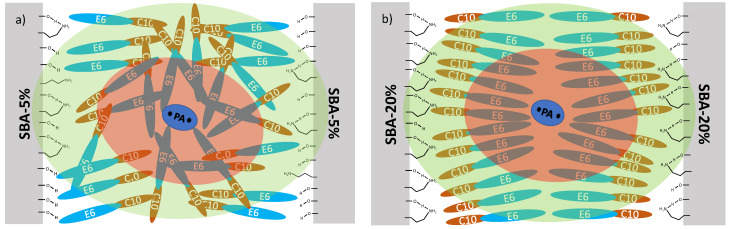
Illustration of (**a**) SBA-5%, (**b**) SBA-20% impregnated with a hydrophilic polarizing agent (PA) in C_10_E_6_. The larger APTES coverage in SBA-20% renders the pore surface more nonpolar than in SBA-5%, which supports the formation of a structured bilayer arrangement of C_10_E_6_ within the pore. The gray blocks represent the silica pore wall, and the red oval area represents the region around the polarizing agent where nuclei cannot be detected by NMR. The green oval represents the region to which nuclei may receive polarization directly from the polarizing agent. Reprinted with permission from Hoffmann et al. [113]. Copyright 2020 American Chemical Society.

In the next step, the effect of surface modifications on the confined surfactants was investigated. In this study, the SBA-15 surfaces were functionalized with APTES at different surface coverage and then impregnated with solutions of DNP radicals (AMUPOL and TOTAPOL) in the non-ionic surfactants polydisperse non-ionic surfactants PEG 200 and C_10_E_6_ as solvent [113]. The samples were characterized by a combination of differential scanning calorimetry (DSC) and DNP-enhanced solid state ^13^C Magic NMR spectroscopy at nominally 120 K. The experiments revealed again the presence of sub-spectra from direct and indirect polarization transfer both for the confined surfactant and for the APTES functionalization. As discussed above, the presence of an indirect polarization transfer process is a clear indication of the local molecular mobility of the observed moiety. Thus, these experiments showed the surprising result that even at 120 K, there are fast dynamics of the APTES functions with correlation times on the order or faster than the inverse Larmor frequency. It was found that the intensities and width of the ^13^C signal MAS NMR spectra were sensitive to the specific combination of radical, surfactant solvent, and APTES surface coverage, showing that this type of experiment can be employed to reveal information about the complex interplay of intermolecular interactions between surface, radical and surfactant and thus the structural organization of the confined system. As discussed in ref. [113] in detail, in the case of amphiphilic C_10_E_6_, there is an interplay of hydrogen bonding and hydrophilic and hydrophobic intermolecular interactions, where the influence of the interactions depends on the surface coverage. At a low surface coverage of 10% (SBA-5%, Figure 8a), there are strong interactions between the remaining polar surface silanol groups and the polar ethylene oxide head group of C_10_E_6_. These interactions, together with interactions with the hydrophilic polarizing agent, compete with the hydrophilic intermolecular interactions between C_10_E_6_ solvent molecules, resulting in a less ordered arrangement of C_10_E_6_. Increasing the APTES surface coverage to 40% (SBA-20%, Figure 8b) reduces the number of possible hydrogen bonds to silanol groups and increases the effect of the hydrophobic interactions, resulting in a high organized bilayer organization of C_10_E_6_ within the pore.

**Figure 9:**
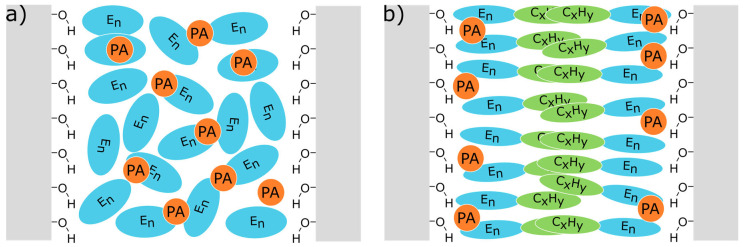
Schematic illustration of (**a**) the hydrophilic surfactants (E5 and PEG 200) and (**b**) the amphiphilic surfactants (C_10_E_6_ and Triton) oriented in the pores of the mesoporous silica host material. E_n_ represents the polyethylene glycol units, C_x_H_y_ represents the lipophilic moiety of the amphiphilic surfactants, and PA represents the polarizing agent AMUPol (not to scale). Reprinted with permission from Döller et al. [124]. Copyright 2023 American Chemical Society.

To evaluate the importance of hydrophilicity in these experiments, Döller at al. [123] performed a study of the behavior of four different commercially available surfactants. The study showed that hydrophilicity is a key factor in the way polarization is transferred from the polarizing agent to the analyte. These investigations laid the foundation to finally perform a systematic comparison between confined hydrophilic surfactants (E_5_ and PEG 200) and confined amphiphilic surfactants (C_10_E_6_ and Triton X-100) in mesoporous silica with DSC and DNP-enhanced solid-state ^13^C MAS-NMR [124]. The samples of SBA-15 and MCM 41 silica materials were impregnated with surfactants. The DNP experiments showed that both the direct and the indirect polarization transfer pathways are active for the carbons of the polyethylene glycol moieties of the surfactants. From the analysis of the spectra, the interactions of the surfactants with the pore walls were determined, and a model of the molecular arrangement of the confined surfactant was proposed (see Figure 9). This model suggests that in the case of the hydrophilic surfactants, all their carbons interact with the silica walls in a similar fashion. In contrast, in the case of amphiphilic surfactants, the terminal hydroxyl group mediates the majority of the interactions with the pore walls and the polarizing agent. The comparison of the different pore sizes showed the surprising result that the influence of the pore size on (SBA 15: 7.0 nm; MCM 41: 4.0 nm) the arrangement of the surfactants in the pore is negligible.

## 4. Surfactants and MD Simulations

MD simulations were applied to surfactants as soon as they became accessible to the study of systems with such a large number of atoms present. First reviews on the simulations of aqueous surfactant solutions appeared as early as 1998 [125,126]. As computational power and computational techniques rapidly improved, the size of systems that can be studied by MD simulation increased congruently. Biomolecules are nowadays routinely simulated, and some topics connecting biomolecules and the topic of surfactants include lipid surfactant drug formulations [127], delivery [128], and the stability of the peptide bilayer membrane [129]. The ability to simulate systems of large sizes has also allowed for the study of how surfactants aggregate [130,131,132,133,134,135,136,137] as well as interact at interfaces of interest such as water oil interfaces, [138,139] CO_2_ foams, [140] carbon nanotubes [141] and liquid–vapor interfaces [142,143]. MD simulations may provide insights into both structural and dynamic details for the system of interest. Introductory reviews on the foundations of MD simulations and best practices are available in the literature [144,145]. Here, we will provide a summary of the fundamental ideas to help readers who are less familiar with MD simulation understand the subject. Where appropriate, specific examples from our research are included.

**Figure 10:**
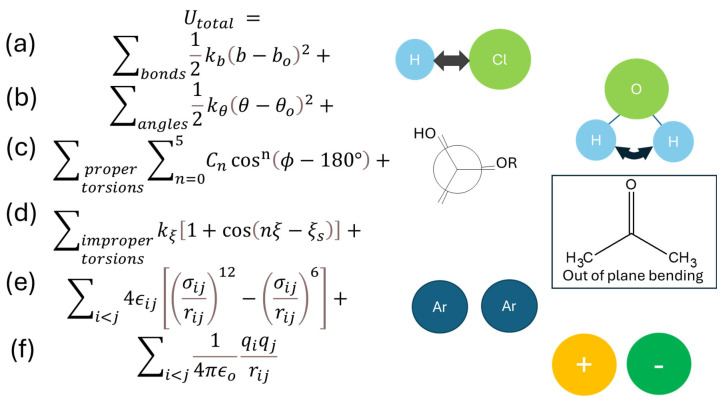
Potential function *U*_total_ consisting of the bonded interactions (**a**–**d**) and nonbonded interactions (**e**,**f**) illustrated schematically by example chemical systems. Bonded interactions describe chemical bond (**a**) and angle vibrations (**b**) by harmonic oscillator functions with force constants $k_{b}$ and $k_{\theta }$ and equilibrium bond lengths/angles $b_{0}$ and $\theta_{0}$, respectively. Proper dihedrals (**c**) may be represented by the Ryckaert–Bellemans potential with torsional energy barrier coefficients $C_{n}$ for n=0, 1, 2, 3, 4, 5 and torsional angle ϕ. Improper dihedrals (**d**) may be represented by a periodic harmonic cosine potential, where ξ is the improper torsional angle at any point in time, $\xi_{s}$ is the phase shift, $k_{\xi }$ is the amplitude of the potential energy curve, and n represents the multiplicity/periodicity of the cosine function. The nonbonded interactions consist of two terms. The Lennard Jones potential represents the London dispersion forces that are present in all chemicals, even noble gases such as argon (**e**), with contact distance, *σ*, and well-depth, *ε*, between two atoms at distance *r* being the interaction parameters. The Coulomb potential function represents the interactions between charges, *q*, where *ε*_0_ is the permittivity of vacuum (**f**).

In an MD simulation, atoms are placed inside a virtual box. In short increments of time steps, such as 2 femtoseconds, they are allowed to move. The direction in which each atom moves depends on the forces to which each atom is exposed. The movements of atoms that are part of a molecule are constraint by the chemical bonds between the atoms of the molecule. Specifically, as illustrated in Figure 10, an atom undergoes (a) a vibrational motion with each other atom it is chemically bonded to, (b) an angular vibration if it belongs to a set of three atoms bonded to each other in nonlinear fashion, (c) a dihedral rotation if it belongs to a set of four consecutively bonded atoms, and (d) an out-of-plane motion (“improper dihedral”) if it belongs to a set of four atoms where three atoms are bonded to a central atom within a plane. In classical MD simulations, the potential functions that govern these so-called bonded interactions are derived from classical physics equations, as shown in Figure 10. In addition to these bonded interactions, there are also the nonbonded interactions typically described by the Coulomb potential and the Lennard Jones potential to account for the interactions between coulombic charges and the London dispersion forces, respectively. The combination of these potential functions is termed the force field as the force, *F*(*r*), is directly obtained from a given potential function, *V*(*r*), by Equation (1).

(1) $$F\left(r\right)=-\frac{dV\left(r\right)}{dr}$$

There are numerous classical forcefields published in the literature, each one having its own unique set of parameter constants and may also vary in detail with respect to the exact form of the used potential equations, especially for the dihedral and improper dihedral potential functions. Some of the more widely used classical force fields have been available for decades and include AMBER, [146,147] OPLS/AA, [148] CHARMM, [149,150] and GROMOS [151]. While the bonding interactions of these force fields are typically based on quantum mechanical ab initio calculations and the parameter sets were optimized for a set of varying molecules against experimental data, foremost densities, they may not describe well other molecules and systems of interest, especially if they are outside of the scope of the types of molecules and systems that were used for the force field development. A more recent approach to improve the accuracy of the MD simulations is to calculate the potentials ab initio at each simulation time step [152]. This ab initio MD (AIMD) method can, at present, only be applied to smaller-sized systems because it is computationally much more demanding than classical MD simulations. A compromise approach is to combine AIMD and classical MD simulations either to verify simulations from classical force fields [153] or to treat only parts of the system with ab initio simulations while simulating the rest of the system classically [154]. On the other hand, if less accuracy is acceptable or atom-level details are not needed, course-graining approaches have been utilized to increase simulation speed and thus access larger systems and/or longer simulation times. For example, the GROMOS force field combines groups of atoms, such as the carbon and hydrogen atoms of a CH_2_ group, into one set of parameters [151], whereas the Martini coarse-grained force field combines entire structural sections [155]. Compared to AIMD, classical MD simulations are also more readily implemented, especially for those force fields where online engines such as LigParGen [156] are available that, upon user input, generate the force field parameter files for the molecule of interest. As an example of the authors’ research efforts that illustrate how a new MD simulation research project may unfold, we desired to better understand polyethylene glycol PEG at the molecular level. PEGs do not possess an amphiphile structure, but they do lower the surface tension of water and are common structural components in non-ionic surfactant molecules, including those illustrated in Figure 1, which have been subject in some of our published research studies [113,116,117,119,122].

A literature search revealed that PEGs were only simulated in aqueous solutions but not in neat form, necessitating own MD simulation efforts. To keep computation costs low, we focused on the commercially available PEG with the lowest average molar weight, which is PEG200. We needed to know its exact composition, which we determined by gas chromatography (GC). GC analysis revealed that the composition does not vary significantly across different sources of vendors and primarily consists of the oligomers di- through heptaethylene glycol [157]. As a first step, we needed to assess the accuracy of available classic force fields in simulating PEG200. We intended to do this first for the neat individual oligomers that make up PEG200 but quickly realized that experimental data on density, self-diffusion coefficient, and viscosity were not available in the literature for the larger oligomers. Thus, we measured these ourselves from 298.15 to 358.15 K for the individual oligomers [118] as well as for PEG200 and PEG400, as here, too, available experimental data were limited [157]. Agreement of simulated density, self-diffusion coefficients, and viscosities with experimental ones decreased with increasing size of the oligomers. The OPLS-AA force field appeared to provide the closest agreement between simulated and experimental data and was used to simulate PEG200. The MD simulations of PEG200 produced viscosities that were about five times larger than the experimental values. We thus explored adjusting some of the force field parameters. Adjusting the polarity of the hydroxy functional groups, as well as lowering the potential energy barrier for the terminal dihedrals, improved the agreement of simulated viscosities to experimental ones [158].

At this point, it is important to note that the implementation of force fields in MD simulations requires close attention to many additional details beyond the force field parameters that need to be followed in accordance with the respective force field for their fair and accurate comparison. MD simulations employ periodic boundary conditions, which in practice means that a molecule moving outside of the box reenters it from the opposite side. Given that potential functions of the nonbonding interactions are only asymptotically approaching zero at large distances, i.e., are non-zero, schemes need to be implemented on how these long-range interactions are accounted for. These schemes typically include a cut-off distance, which needs to be set to a value as prescribed by those who published the force field. There are also various schemes for equilibrating the simulation box with respect to temperature and pressure. These general simulation details are nicely explained and reviewed by Braun et al. [144]. Other details of an MD simulation depend on the goal of the simulations. For example, to properly obtain self-diffusion coefficients, the simulation needs to be long enough for the system to reach the diffusive regime. Furthermore, the center of mass of the simulation box may drift during a simulation, which needs to be corrected during the simulation, and the self-diffusion coefficients are box-size dependent, for which known correction expressions exist [159]. Viscosities, on the other hand, require frequent enough sampling of the system energies and sufficient statistics. These and other best practices for MD simulations to obtain accurately simulated self-diffusion coefficients and viscosities are carefully described by Maginn et al. [160]. Finally, classical simulations may inherently fall short of obtaining some system properties accurately. Specifically, there are quantum effects that may not be captured in classical simulations that inherently lead to molar heat capacities that are about two times as large as the actual experimental values [161].

Despite these just descript pitfalls of classical MD simulations, they provided us with the sought answer to the initial question about PEG200. The oligomer components in PEG200 are dispersed evenly, so PEG200 should be thought of as a homogeneous mixture of ethylene glycol oligomers [158]. Furthermore, the simulations confirmed a prior made experimental observation that a binary mixture of tri- and hexaethylene glycol with the same average molar mass of 200 g·mol^−1^ has the same physical properties of density, self-diffusion coefficient, and viscosity. Apparently, the specific makeup of the ethylene glycol oligomer mixture is not important, so the physical properties are only governed by the average molar mass [157,158]. The PEG200 MD simulations also provided valuable molecular-level insights. The adjustments of the OPLS-AA force field to improve agreement between simulated and experimental properties led to considerably fewer hydrogen bonding interactions with a concurrent shift from inter- to intramolecular hydrogen bonding, which shows how sensitive the physical properties are to these intermolecular interactions. Moreover, tetraethylene glycol showed an extraordinarily high propensity towards intramolecular hydrogen bonding (see Figure 10). This has led us to our current, still ongoing investigation of the interplay between inter- and intramolecular hydrogen bonding in simpler systems, namely 1-octanol related ether alcohols that can form only one type of hydrogen bonding each: intermolecular hydrogen bonding between the hydroxy groups, intermolecular hydrogen bonding between hydroxy and ether functional groups, and intramolecular hydrogen bonding between hydroxy and ether functional groups. Here, experimental data also needed to be acquired first to test the accuracy of the force fields [162]. These experimental data indicate that intramolecular hydrogen bonding is most prevalent when the ether functional group is in an optimum number of covalent bonds away from the hydroxy group. The presence of inter and intramolecular interactions also prevents the formation of a crystalline solid upon cooling [163].

Right panel: Radial distribution functions obtained with the TIP4P/2005 and OPLS forcefields of water oxygen with hydroxy (black) and with ether oxygen (red) of hexaethylene glycol, each from four different water mass fractions of 0.001, 0.005, 0.010, and 0.020. Aside from different noise levels, the respective radial distribution functions are indistinguishable.

**Figure 11:**
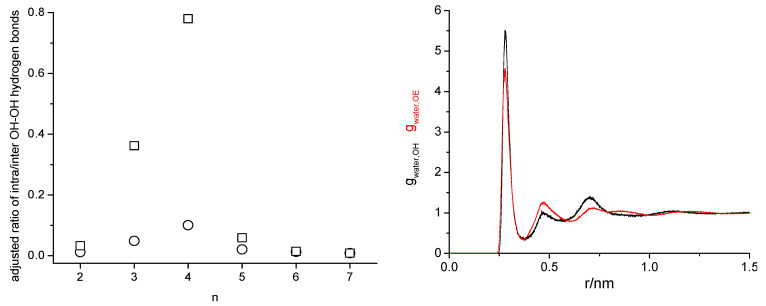
**Left panel**: Adjusted ratio of intra-over intermolecular hydrogen bonds between hydroxy hydrogen and hydroxy oxygen for each oligomer with itself in PEG200 obtained from MD simulations at 328 K using the OPLS force field (circles) and modified OPLS force field (squares). The ratio numbers were adjusted by the number of possible intra- and intermolecular hydrogen bonds, the respective oligomer mole fraction, and a scaling factor depending on the number of oligomer components. Reprinted with permission from Hoffmann et al. [158]. Copyright 2023 American Chemical Society. **Right panel**: Radial distribution functions obtained with the TIP4P/2005 and OPLS forcefields of water oxygen with hydroxy (black) and with ether oxygen (red) of hexaethylene glycol, each from four different water mass fraction of 0.001, 0.005, 0.010, and 0.020. Aside from different noise level, the respective radial distribution functions are indistinguishable.

Overall, classical MD simulations are especially helpful in answering qualitative questions. The answers obtained from classical MD simulations need to be confirmed from simulations using different force fields to ensure that they are not artifacts from a specific force field. We took this approach also in our recently completed investigation on PEG 200 and some of its neat oligomer components, where we sought an answer to the question of why, as we observed experimentally, present water impurities hardly affect their physical properties, up to mole fractions of about 0.15 [164]. We used two different force fields for the ethylene glycol oligomers for this study, the OPLS-AA, and the OPLS-AA forcefield, with our prior modifications, as well as two different force fields for water, the SPCE and the TIP4P/2005 force field. These all reproduced the same experimentally observed trend of water independence up to 0.15-mole fractions as exemplary illustrated by the water content independent radial distributions are shown in Figure 11 and revealed that this observation is likely due to competing effects of water disturbing the interplay of inter- and intramolecular hydrogen bonding in PEG200 [164].

With respect to MD simulations of surfactants in porous media, there is rather little published overall, leaving the field wide open. One research area, though, where MD simulations have frequently been used, concerns the application of extracting remaining oil in reservoirs. This process typically involves the use of surfactants to solubilize and detach the remaining oil from the soil surface, including asphaltenes, which are the heaviest fractions of bitumen. As a model surface to represent the rock type of the oil reservoir, the simulations often use quartz, but other surfaces have been simulated as well, such as carbonate. Ahmadi and Chen nicely summarize in tabular form the MD simulation outcomes from prior work in their 2022 article [165]. These combined simulation outcomes show how much mechanistic detail MD simulations can reveal on how the surfactant, in a competitive way, interacts with various aliphatic and aromatic components of the bitumen as well as the solid surface. These insights lead to practical recommendations such as for example, how far an increase in pumping force can improve the detachment process of the oil from the surface [165]. On the same subject, a very recent combined experimental and MD simulation study focuses on hydrolyzed polyacryl amide strengthened nitrogen foam (HSNF) to recover the remaining oil in sandstone. These MD simulations consisted of oil, water, nitrogen, surfactant, and polymer confined in silica pores to emulate sandstone. The obtained radial distribution functions, self-diffusion constants, and interaction energies obtained from these simulations showed that the addition of the surfactant and polymer resulted in a more uniform distribution of water and nitrogen throughout the pore by avoiding that water and nitrogen come into direct contact [166]. Ahmadi et al. point out that coarse-graining approaches, such as dissipative particle dynamics (DPD), are needed to be able to expand simulations to systems that consist of more than a million atoms [131].

Other applications involving confined surfactants where MD simulations were employed to obtain a molecular-level understanding include improving the performance of concrete, [167] electrochemical storage devices, [168] and the environmental impact of functionalized multi-walled carbon nanotubes [169].

Concrete such as ordinary Portland cement (OPC) is a widely used construction material. Improving its properties by incorporation of additives such as nanoparticles may, therefore, have an immensely positive impact on society. One such nanoparticle material is boron nitride nanosheets (BNNS) due to its tightly packed two-dimensional honeycomb structure that gives rise to excellent mechanical strength. Surfactants play a key role during the mixing of cement with water and following curing processes. Ranjkesh et al. report a combined experimental and MD simulation study to better understand these suspensions [167]. They showed that BNNS by itself is dispersible in water but cannot maintain the dispersity in the simulated pore solution. From the ions that are present in OPC, Ca^2+^ is the main cause of BNNS agglomeration. The surfactants significantly improved the dispersion stability of the BNNS within the simulated pores.

Surface active ionic liquids (SAILS) are ionic compounds where one of the ions is either an anionic or cationic surfactant ion, thereby combining the benefits of being a surfactant as well as an ionic liquid. Zhang et al. studied several types of cationic and anionic SAILS by MD simulations where the SAILs were confined between plates of opposite charge [168]. The surface alignment of the SAILs and the increase of the SAIL number density near the surface were strongly dependent on their charge and structural characteristics.

Carbon nanotubes (CNTs) are increasingly used in important applications such as sensors, electronics, and hydrogen storage. CNTs tend to aggregate in aqueous media, which is undesirable for some industrial and environmental applications. Biodegradable non-ionic surfactants are preferably used to stabilize CNTs [169]. Zhang et al. present a combined experimental and MD simulation study of carbon nanotubes stabilize in water by the surfactant Triton X-100 in quartz sand [169]. These MD simulations confirmed the experimentally observed trends that the surfactant helps in retaining the CNT in the pore only if the surfactant concentration is below about 50 mg·^−1^, while higher surfactant concentrations cause the surfactants themselves to aggregate, which in turn deteriorates the CNT suspensions.

Finally, there is the more fundamental research subject of how surfactant solutions are taken up into porous material. Absorption of C_m_E_n_ non-ionic surfactants from aqueous solutions in hydrophilic mesoporous silica has been studied experimentally and by molecular modeling [55,56,57,59,170,171,172,173,174,175,176,177]. Combined, these studies show that increasing surfactant concentration changes the surfactant adsorption from small patches to micelles, to worm-like aggregates, and eventually to bilayer formation. A subset of these studies also investigated the temperature dependence of the absorption process under consideration that the water-C_m_E_n_ binary system separates into two liquid phases above a lower critical solution temperature (LCST) [55,56,57,59,170]. The most recent report we are aware of is a combined experimental and MD study of aqueous solutions of C_6_E_3_ in SBA-15, where it is shown that at temperatures above the LCST, the surfactant is taken up into the pores and adsorbs on the surface as micellar aggregate at sufficiently high surfactant concentrations. The reverse is observed for temperatures below the LCST, giving rise to the conceptual design of materials with temperature-induced uptake and release capabilities [59].

## 5. Conclusions

The present review gives an overview of current trends in the application of solid-state NMR and Molecular Dynamics simulations for the investigation of surfactants of various sizes and complexities in confined environments. It is shown that the approach to combine multinuclear solid-state NMR techniques with signal enhancement techniques like DNP, thermodynamic techniques like calorimetry, and molecular dynamics simulations provides important insights into the phase behavior and arrangement of the surfactants and their interactions with the confining hosts. As demonstrated by the variety of surfactants ranging from small model molecules like octanol to technical PEG mixtures, the presented approach is not limited to simple model systems but can also be applied to “real world” systems. This opens up perspectives for future applications for the characterization of confined surfactants and their ubiquitous applications in commercial products.
